# A non-randomized clinical trial to determine the safety and efficacy of a novel sperm sex selection technique

**DOI:** 10.1371/journal.pone.0282216

**Published:** 2023-03-22

**Authors:** Stephanie Cheung, Rony Elias, Philip Xie, Zev Rosenwaks, Gianpiero D. Palermo

**Affiliations:** The Ronald O. Perelman and Claudia Cohen Center for Reproductive Medicine, Weill Cornell Medicine, New York, New York, United States of America; ANZAC Research Institute, AUSTRALIA

## Abstract

The desire to have offspring of a specific sex has a long history but has been particularly present with the appearance of assisted reproduction. However, embryo selection raises ethical concerns. Thus, several techniques to select sex-specific spermatozoa have been proposed but carry limitations. There are many variations of each technique, and some are time consuming and costly. Concerns about effectiveness and safety have also rendered many of them unappealing. Therefore, we propose a novel sperm sex selection technique (SST) that appears to be consistently safe and effective. A single-center, non-randomized clinical trial was designed. We included 1,317 couples, who were assigned to one of two groups: ICSI/PGTA or ICSI/PGTA+GS. Ejaculates from male partners of couples in the ICSI/PGTA+GS group (n = 105) were processed using SST to enrich spermatozoa for their desired sex. Standard sperm processing was carried out for couples undergoing PGT-A solely for aneuploidy (n = 1,212), comprising the ICSI/PGTA control group. To validate the efficacy of our technique, we performed an analysis on spermatozoa pre- and post-selection, followed by an assessment of the proportion of the conceptuses’ sex to confirm clinical reliability. We also followed up on ICSI clinical outcomes and child/newborn health to establish the safety of our method. Our main outcome measures included the proportion of spermatozoa and embryos enriched for female and male sex, as well as embryo euploidy rates and ICSI clinical outcomes. These outcomes were compared between the two groups. For the ICSI/PGTA group (n = 1,212) (maternal age, 37.0±4yrs; paternal age, 39.1±6yrs), with ejaculated spermatozoa processed in the standard fashion, 2,303 ICSI cycles (1.2±1) yielded an 81.0% (14,375/17,737) fertilization. PGT-A results indicated a euploidy rate of 73.1% (n = 3,718) for female and 72.4% (n = 3,054) for male embryos. These couples achieved a 76.4% (699/915) implantation and 65.2% (597/915) clinical pregnancy rate, with 551 deliveries (48.5% female, 51.5% male). All 105 men in the ICSI/PGTA+GS group had sperm specimens with an equal sex distribution at baseline. Of them, 59 (paternal age, 40.9±6yrs) who desired female offspring obtained an 81.6% enrichment after SST. They underwent 73 ICSI cycles with their partners (maternal age, 37.9±4yrs), achieving a 77.3% (583/754) fertilization. This resulted in 79.1% (231/292) female embryos that generated a 79.3% (23/29) implantation rate, with 16 singleton deliveries of the desired female sex without major or minor congenital malformations. Forty-six couples (maternal age, 37.3±4yrs; paternal age, 40.7±6yrs) desiring male offspring obtained an 80.8% sperm sex enrichment. They underwent 50 ICSI cycles, achieving a 75.4% (462/613) fertilization and equivalent proportion of male embryos (223/280, 79.6%). Their implantation was 90.5% (19/21), with 13 singleton deliveries of healthy male offspring. Furthermore, 78.8% (182/231) of female and 66.4% (148/223) of male embryos from the ICSI/PGTA+GS cohort were euploid. These euploid rates were comparable to those from the ICSI/PGTA group. In couples undergoing ICSI with PGT-A, SST consistently enriched spermatozoa, resulting in a higher proportion of embryos and thus offspring of the desired sex. Moreover, SST did not impair the fertilization or embryo developmental competence of spermatozoa, nor did it affect offspring health.

**Trial registration:** Clinicaltrials.gov NCT05500573.

## Introduction

The desire to have offspring of a specific sex has a long history but has been particularly present since the 1970s with the early appearance of assisted reproduction. The reasons for choosing a child’s sex may be social, such as a desire for family balancing [1]. Couples undergoing IVF, who already have a child or children of one sex, may wish to have the experience of raising children of both sexes. Some couples, who already have children, could have financial reasons for not attempting a further pregnancy without assurance that the additional child will be of a specific sex. Preference for the sex of a given offspring may also reflect on a couple’s cultural belief that there are differences between the experience of raising male and female children [2]. There are also health-related reasons for choosing a child’s sex, such as the need to avoid sex-linked diseases [3]. If the disease itself is X-linked, there is a need to determine the embryo sex to avoid transmission of an affected male embryo. Although a preference for offspring of a specific sex is common, embryo selection may raise ethical concerns. Thus, over the years, several techniques to select sex-specific spermatozoa using disparate methods, including Albumin and Percoll gradients, swim-up methods, gel filtration, flow cytometry, and even electrophoresis, have been proposed [4–9].

Swim-up–based techniques allow spermatozoa selection without the use of centrifugation. For this method, the sperm specimen is placed in a tube, and a culture medium is carefully layered on top. In some instances, the tube is placed at a 45-degree angle at room temperature, or at 37°C. Spermatozoa are then allowed to swim up into the culture medium for approximately one hour. For female sex selection, the top layer is collected for use. For male sex selection, a modified technique is used where a small fraction of the top layer is first discarded, and the bottom portion is retrieved instead. However, there have been conflicting reports on the efficacy of this method. Some studies have observed a >70% sex enrichment [10], while others have found that the spermatozoa sex ratio remains unaffected [11]. Furthermore, little seems to have been published on the sex proportion of conceptuses generated from spermatozoa selected by this method.

Unlike the swim-up selection method, which is dependent on sperm density, free flow electrophoresis selects for sex-specific spermatozoa according to net charge. A buffer is circulated through the chambers by electrodes while the semen specimen is deposited into the chamber continuously. This method was originally observed to provide a 90% enrichment for female and an 80% enrichment for male [12]. However, later studies were unable to replicate these results, instead concluding that the movement of spermatozoa toward the anode may be due to their surface sialic acid content rather than their genotype [9,13]. As a result, the application of this method in a clinical setting has been limited.

Another approach, utilizing a Sephadex gel filtration, separates cells by size. Supposedly, lighter cells, such as male spermatozoa, are easily trapped, whereas those of larger molecular weight, X-bearing spermatozoa, can easily pass through. Therefore, although this technique has been shown to achieve an 80% accuracy for the proportion of female spermatozoa [14], Y-spermatozoa selection did not appear feasible. Also, there is no literature confirming the efficacy of this method.

The Percoll process employs discontinuous gradient layers at varying concentrations, where the semen specimen is layered on top of the gradients and centrifuged. This technique is effective at increasing overall sperm motility, but when applied for sperm sex selection, it has yielded inconsistent results [15,16]. Unlike the aforementioned methods, this technique has been tested more thoroughly, albeit mainly in animal models and yielding modest results. For instance, when applied in bovine spermatozoa, this technique generated embryos that were found to have only a 60% skewing, at best, toward the selected sex [5].

Ericsson’s Albumin Method is performed by layering aliquots of the sperm specimen on human serum albumin in a column, and then removing the individual layers at specific time intervals. An enrichment of female and male spermatozoa with 75% and 85% accuracy, respectively, was achieved with the use of this technique [17]. Furthermore, this sex selection method is one of the few that have been tested in couples undergoing IVF cycles with PGT-A. However, it has been demonstrated that increasing spermatozoa of a specific sex does not translate to a corresponding proportion of embryos tested [18].

Perhaps the most effective, yet controversial, method of sperm sex selection is flow cytometry. With this technique, sperm cells are exposed to fluorescent dyes that label their genetic material. Since X-bearing spermatozoa have 2.8% more genetic material, they fluoresce brighter, thereby differentiating them from Y-bearing spermatozoa. This method, capable of achieving a 90% enrichment for X- and 80% enrichment for Y-bearing spermatozoa [8], is common for sperm sex selection in animal husbandry [19]. However, the quality of the resulting spermatozoa is dependent on the species. For instance, previous data have shown that boar spermatozoa are more susceptible to membrane damage during flow cytometry, with consequent impairment of fertilization and embryo development [20]. Furthermore, although attempts have been made to address these drawbacks, such as adjustments of laser power and the types of fluorescent dyes, none have been definitively successful [21–23]. This, among other reasons, led to the discontinuation of the commercially available technique known as MicroSort^®^.

These methods all have advantages and limitations. There have been many variations of each technique, specifically related to the timing of centrifugation and the concentrations of gradient layers. Some methods, in addition to being time consuming, require costly equipment, such as a cell sorter. Furthermore, although there have been several reports on the efficacy of these techniques, there have also been studies disputing their effectiveness [24,25]. In particular, the exposure of spermatozoa to fluorescent dyes, laser light, and electrical charges has raised concerns about the possibility of these techniques contributing to DNA damage, thus rendering many of them unappealing and obsolete.

Based on the limitations of these techniques, we propose a novel sperm sex selection method that appears to be consistently safe and effective. Moreover, since very few studies have assessed, for a single method, the technical efficacy of skewing a sperm specimen toward a specific sex, the clinical reliability of generating embryos of that desired sex, and the method’s impact on offspring health, we aimed to conduct a thorough evaluation of our novel method that addresses each of the aforementioned aspects in order to propose a valuable, feasible, and effective method to provide couples with a higher proportion of embryos of their desired sex. In addition to the medical rationale of avoiding sex-linked diseases or family balancing, the scientific rationale for this sperm-based sex selection technique is to introduce an ethical method to successfully skew the proportion of embryos towards a couple’s desired sex without impairing embryo developmental competence or offspring health, while minimizing embryo wastage. For couples undergoing intracytoplasmic sperm injection (ICSI), we performed a fluorescent in situ hybridization (FISH) assessment on spermatozoa pre- and post-selection to validate the efficacy of our technique. Our analytical plan also included an assessment of the proportion of the conceptuses’ sex to confirm clinical reliability. We also followed up on ICSI clinical outcomes and child/newborn health to establish the safety of this sex selection method.

## Materials and methods

### Inclusion criteria and study design

**Fig 1:**
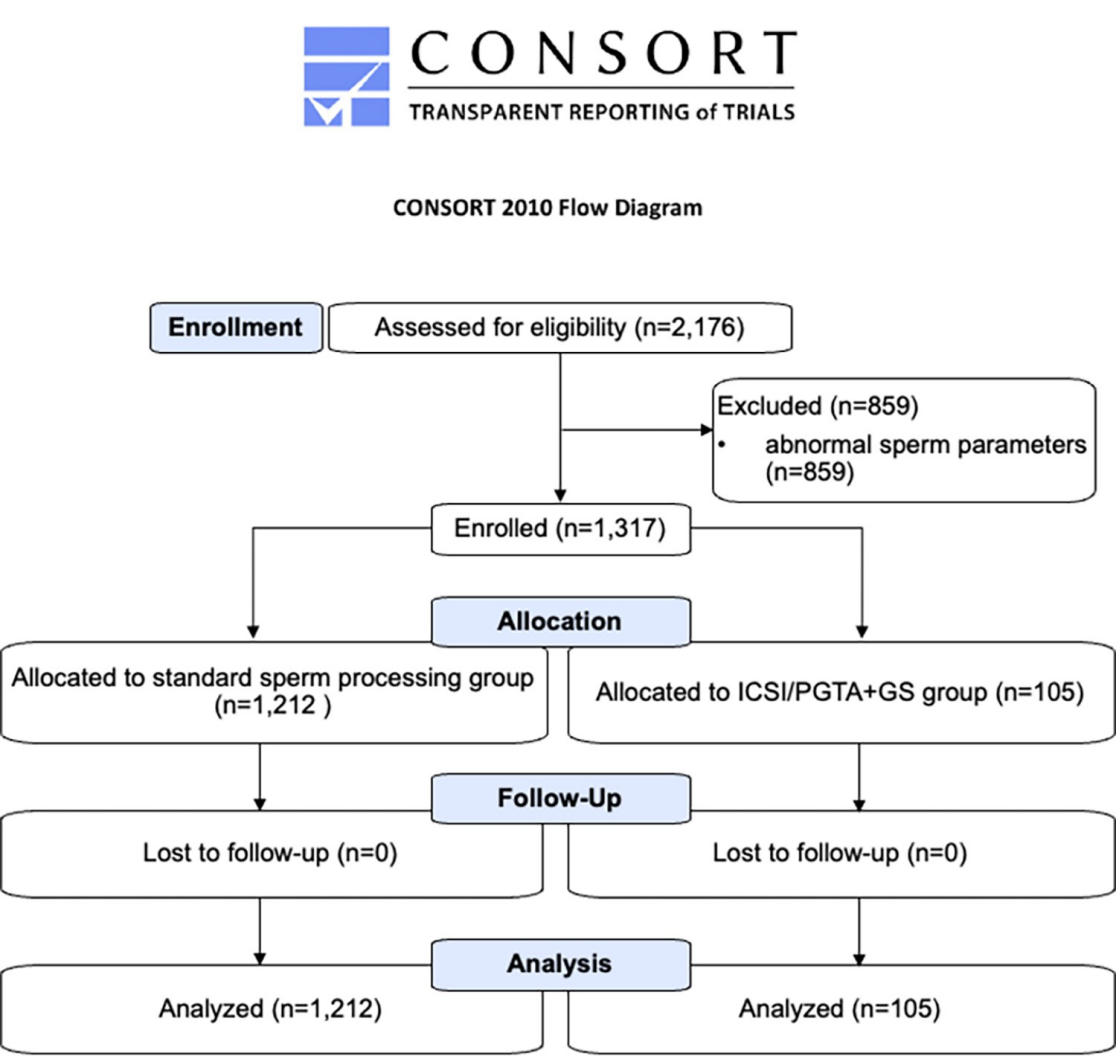
CONSORT flow diagram.

From August 2016 to July 2020, a total of 1,317 consenting couples undergoing ICSI cycles at our center with preimplantation genetic testing either for aneuploidy (ICSI/PGTA) or interest in a specific offspring sex (ICSI/PGTA+GS) were included in our study (Fig 1). The Institutional Review Board of the New York-Presbyterian Hospital–Weill Cornell Medicine approved this study (IRB 1306014043), and all patients gave informed written consent to participate. This study was also registered at ClinicalTrials.gov (NCT05500573) once clinical outcome data was available for ICSI/PGTA couples. The authors confirm that all ongoing and related trials for this intervention are registered.

Male partners were first screened by a semen analysis. We also carried out a genetic assessment on spermatozoa from the raw ejaculates by fluorescent in situ hybridization (FISH) to determine the proportion of X- and Y-bearing spermatozoa prior to sperm processing. Standard sperm processing was performed for the control group, consisting of 1,212 couples undergoing ICSI with PGT-A solely for aneuploidy. For 105 consenting couples, sperm specimens were enriched for their desired sex. The processed specimens were subsequently used in their ICSI cycles with PGT-A, and a small portion was retained for a blinded assessment by FISH. The proportion of X- to Y-bearing spermatozoa, as well as ICSI and PGT-A outcomes, were compared between the two cohorts. Our main outcome measures included the proportion of spermatozoa and embryos enriched for female and male sex, as well as embryo euploidy rates and ICSI clinical outcomes.

### Spermatozoa collection and preparation

For the 1,212 couples undergoing ICSI with PGT-A solely for aneuploidy, semen samples were collected by masturbation after 2–5 days of abstinence and allowed to stand for 15 minutes at 37°C to promote liquefaction. Concentration and motility were analyzed by viewing 5 μL of the semen sample in a Makler chamber. The sample was then combined with media comprised of HEPES-buffered human tubal fluid (H-HTF; Irvine Scientific, CA, USA) supplemented with human serum albumin (HSA solution G Series culture media; Vitrolife, Goteborg, Sweden) and centrifuged at 600 *g* for 10 min. The pellet was then resuspended and evenly layered onto density gradient (Enhance-S Plus Cell Isolation Media, 90%; Vitrolife, Goteborg, Sweden) and centrifuged a second time at 300 *g*. Next, the bottom layer containing the motile spermatozoa was aspirated using a glass Pasteur pipette and resuspended in medium for a final centrifugation at 600 *g* for 10 min to remove the silica gel particles from the sample. The sample was brought down to a volume of 0.5 mL and resuspended. The processed sample was assessed for concentration and motility and used for ICSI.

### Multilayer density gradient sperm selection

**Fig 2:**
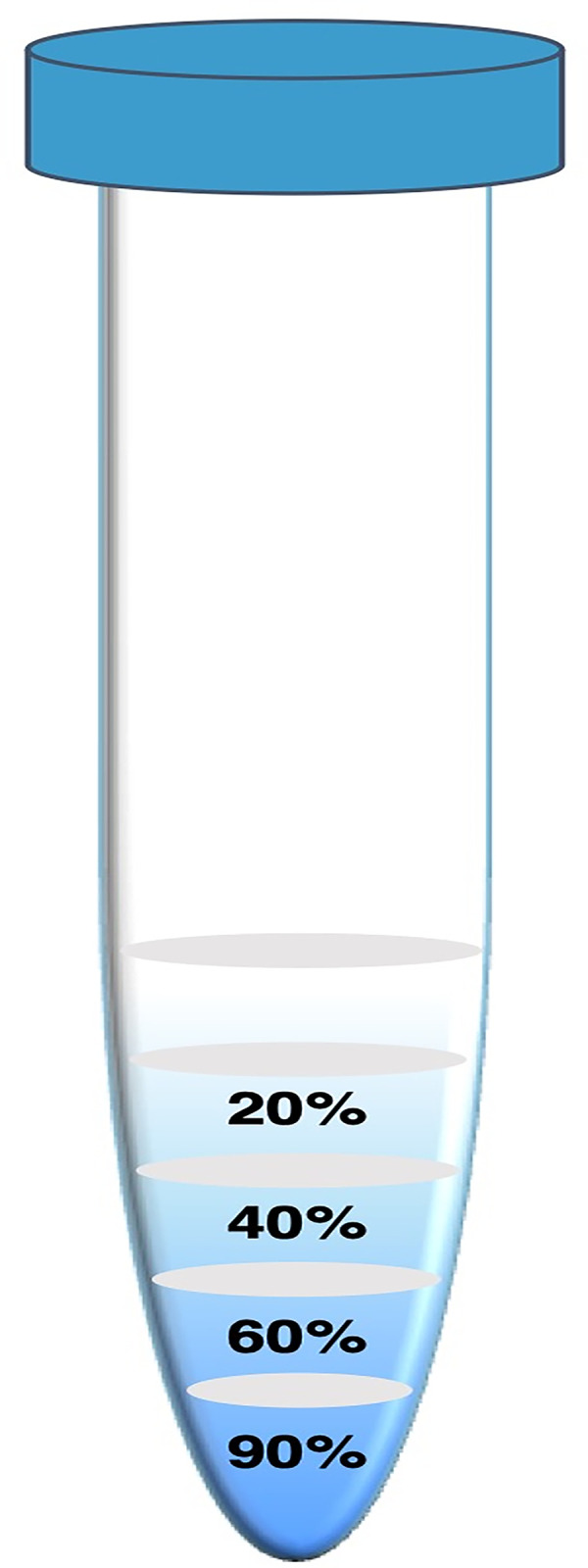
Sperm sex selection protocol. For the study cohort, ejaculates were processed by a multilayer density gradient (Enhance-S Plus Cell Isolation Media, 100%; Vitrolife, Goteborg, Sweden) comprised of four concentrations: 20%, 40%, 60%, and 90%. After removing the seminal fluid, the resuspended sperm pellet was layered on top, and spermatozoa were able to self-select for 90 minutes at 4°C. Y-bearing spermatozoa were retrieved from the upper, 20% layer, and heavier X-bearing spermatozoa were retrieved from the bottom, 90% layer.

For 105 couples undergoing ICSI with PGT-A for sex selection, ejaculates were processed by an in-house protocol that utilized a multilayer density gradient (Enhance-S Plus Cell Isolation Media, 100%; Vitrolife, Goteborg, Sweden) comprised of four concentrations: 20%, 40%, 60%, and 90% (Fig 2). After testing a variety of density gradients and incubation times [26], this particular formation was found to yield the most accurate and consistent results in providing the highest gender purity, and was therefore selected. After removing the seminal fluid, the resuspended sperm pellet was layered on top, and spermatozoa were able to self-select for 90 minutes at 4°C. Y-bearing spermatozoa were retrieved from the top density gradient layer, and heavier X-bearing spermatozoa were retrieved from the bottom, 90% layer. The selected portions were resuspended in medium for a final centrifugation to remove silica gel particles. The specimen was then brought down to a 0.5-mL volume and resuspended. The final concentration and motility were assessed, and the sample was subsequently used for ICSI.

### Preparation of spermatozoa for FISH analysis

Slides were fixed in Carnoy’s fixative (3:1 methanol:acetic acid) at room temperature for 15 minutes and placed on a 37°C slide moat overnight. Sperm decondensation was achieved by placing slides into a Coplin jar containing 10 mmol/l dithiothreitol (DTT; Sigma Chemical Co., St. Louis, MO, USA) in 100 mmol/L tris(hydroxymetyl) aminomethane (Trizma HCl; Sigma Chemical Co.) at 22°C for 3 minutes. Slides were then washed for 1 minute in 2x standard saline citrate (SSC; Vysis, Downers Grove, IL, USA) and hybridized with fluorescent probes. Negative controls were included in each assessment and processed identically, except for the probe hybridization step [27]. Positive controls were set by processing slides smeared with somatic cells of known gender. Seven μl of 4’,6-diamino-2-phenylindole (DAPI; Abbott Molecular, Des Plaines, IL, USA) was used to counterstain sperm nuclei. Each slide was then cover-slipped and assessed on a fluorescent microscope (Olympus BX61; New York/New Jersey Scientific, NJ, USA) at 1,000x. Slides were processed and assessed in replicate to reduce FISH error, and at least 1,000 cells per patient were assessed to determine the ratio of X:Y spermatozoa (Applied Imaging, CytoVysion v3.93.2). It should be noted that although we maintain a 2–3% FISH error when conducting a typical sperm aneuploidy assessment, the FISH error for confirming sperm sex enrichment was negligible (<1%).

### Ovarian superovulation and oocyte collection

To determine the stimulation protocol, patient age, weight, antral follicular count, serum anti-Müllerian hormone (AMH) level, and previous response to stimulation were carefully examined [28]. Patients were treated with daily gonadotropins (Follistim, Merck, Kenilworth, NJ, USA; Gonal-F, EMD-Serono, Geneva, Switzerland; and/or Menopur, Ferring Pharmaceuticals Inc, Parsippany, NJ, USA), and pituitary suppression was achieved by GnRH-agonist (leuprolide acetate, Abbott Laboratories, Chicago, IL, USA) or GnRH-antagonist (Ganirelix acetate, Merck, Kenilworth, NJ, USA; or Cetrotide, EMD-Serono Inc., Rockland, MA, USA). To attain follicular synchronization, some patients were treated with oral contraceptive pills (Ortho-Novum, Janssen Pharmaceuticals, Beerse, Belgium) before starting gonadotropins. The human chorionic gonadotropin trigger (hCG, Ovidreal, EMD Serono) for final oocyte maturation was administered when at least 2 lead follicles reached an average diameter of ≥17 mm. Transvaginal oocyte retrieval was performed under conscious sedation 35–37 hours after hCG administration. Oocytes were further incubated for an additional 3–4 hours post-retrieval. Prior to micromanipulation, the cumulus-corona cells were removed by exposing the oocytes to medium containing 40 IU/mL of hyaluronidase (Cumulase, Halozyme Therapeutics, Inc. San Diego, CA) [29,30]. To facilitate this process, oocytes were aspirated in and out of a calibrated pipette stripper (Origio, Målov Denmark) with an approximately 200-μm inner diameter. Complete removal of the adhering corona radiata was necessary to prevent visual obstruction of the oocyte as well as the holding and/or injection pipettes by the residual corona cells. Each oocyte was washed twice in culture medium (home-brew, modified Cornell medium based on G1 and G2 components; Vitrolife, Sweden) [31,32] and then examined under an inverted microscope (TE2000U, Nikon USA, Melville, New York, USA) equipped with 2x, 4x, and 10x objectives (Nikon CFI Apo), and 20x and 40x objectives (Nikon Polarized optics CFI Plan Fluor) to assess integrity and maturational stage. After cumulus removal, oocytes at prophase I displayed a germinal vesicle, and at metaphase I, the germinal vesicle breaks down (GVBD) without extrusion of the polar body (PB). Once the first PB was identified, oocytes were considered to be at the MII stage and ready for ICSI.

### Embryo culture and morphologic and cytogenetic evaluation

Embryo evaluation, criteria, and the biopsy procedure have been described previously [33]. Day-3 embryos considered good quality had morphologic grades of 1–2 (≥8 cells with even or slightly uneven blastomere expansion and ≤20% fragmentation). Day-5 good-quality embryos had the following morphologic grades: blastocele, 1–3 (degree of expansion ≥50% the volume of the embryo); inner cell mass, A–B (clear inner cell mass with healthy cells); and trophectoderm, A–B (healthy cells). The biopsy procedure was performed on day 5 as follows: The embryo was positioned by holding a pipette while laser pulses (ZI-LOS-tk Laser) were used to create an opening in the zona pellucida to allow for aspiration of 3–7 trophoblastic cells with a 20-μm-diameter biopsy pipette. Cells were transferred in 200 μL of polymerase chain reaction tubes with 2 μL of lysis buffer and analyzed by array comparative genome hybridization (Bluegnome 24SureV3 chip; Illumina, San Diego, CA). Biopsied embryos were rinsed in a culture buffer and then vitrified [34] until the cytogenetic results were available and patient synchronization was achieved for embryo transfer.

### Embryo transfer and pregnancy assessment

Patients received intramuscular progesterone supplementation (50 mg daily) starting 1 day after retrieval. Couples underwent a fresh embryo transfer on day 3 or 5 according to the developmental characteristics of the embryo. Couples that underwent PGT-A had euploid embryo transfers in natural or programmed frozen embryo transfer (FET) cycles [35]. The endometrial lining had to be at least 7 mm for a patient to be considered for an embryo transfer. Serum βhCG levels were measured 14 days after retrieval. Implantation rate was defined as number of gestational sacs out of the total number of embryos transferred. Clinical pregnancy was defined as the detection of an intrauterine implantation sac with fetal heart activity on ultrasound. Information on delivery method and neonatal wellness conditions was obtained from the couples’ obstetrician and pediatrician, as well as from their records. Follow-up on child development was also offered at 3 years of age, using parent-administered questionnaires as previously described [36].

### Statistical analysis

Spermatozoa enrichment and PGT-A outcomes were compared between the ICSI/PGTA and ICSI/PGTA+GS groups using unpaired t-test (Stata Statistical Software, StataCorp LLC, College Station, TX). Friedman’s Chi-Square and Fisher’s Exact tests (Jandel Scientific, San Rafael, CA) were used to compare the ICSI cycle outcomes between the control and sex selection cohorts. *P* values <0.05 were considered statistically significant.

## Results

**Fig 3:**
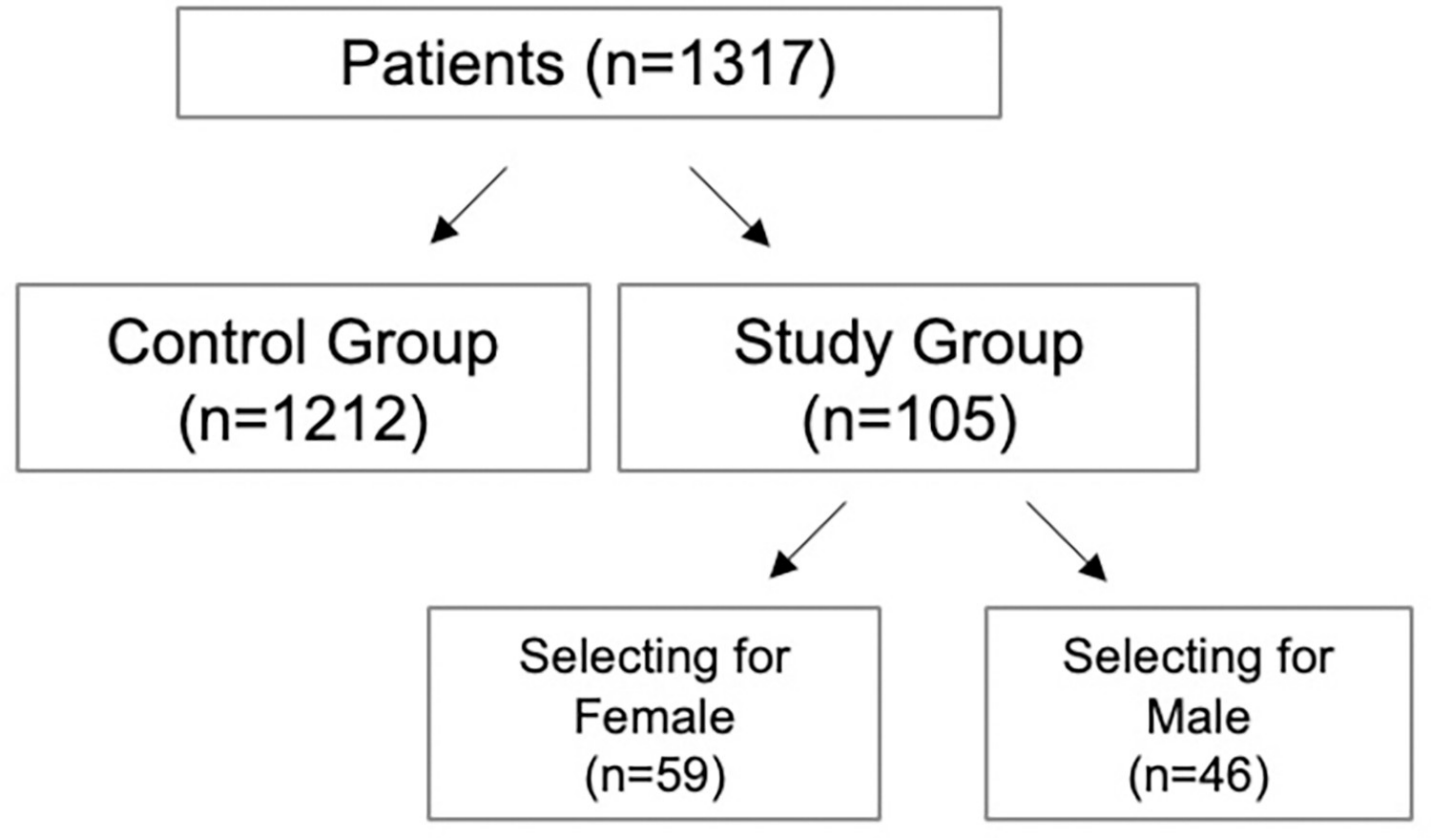
Patient flowchart. A total of 1,317 couples undergoing ICSI cycles with preimplantation genetic testing were included in our study. Couples (n = 1,212) who did not desire a specific offspring sex underwent ICSI cycles with PGT-A solely for aneuploidy, while the remaining 105 elected to undergo ICSI with PGT-A for sex selection.

Overall, 2,176 couples were assessed for eligibility from August 2016 to July 2020. Those with abnormal sperm parameters (n = 859) were excluded. A total of 1,317 couples (maternal age, 37.1 ± 4 yrs; paternal age, 39.2 ± 6 yrs) undergoing ICSI cycles with preimplantation genetic testing were enrolled in our study (Fig 3). Overall sperm concentration was 53.8±37x10^6^/mL, with 40.5±14% motility and 3.1±1% normal morphology. FISH assessment performed on the raw ejaculates confirmed an even distribution of X- (49.4±2%) and Y- (50.7±2%) bearing spermatozoa.

Couples (n = 1,212) who did not desire a specific offspring sex underwent ICSI cycles with PGT-A solely for aneuploidy (ICSI/PGTA), while the remaining 105 elected to undergo ICSI with PGT-A for sex selection (ICSI/PGTA+GS) (Table 1).

**Table 1:** Demographics and semen parameters for the control and study groups according to sex.

		ICSI/PGTA+GS	
	ICSI/PGTA	Female	Male
**Couples**	1212	59	46
Maternal age (M yrs±SD)	37.0±4	37.9±4	37.3±4
Paternal age (M yrs±SD)	39.1±6	40.9±6	40.7±6
***Semen Parameters***			
Concentration (10^6^/mL±SD)	47.8±14^ab^	16.7±15^a^	27.0±25^b^
Motility (%±SD)	87.9±12	88.5±11	83.8±14
Morphology (%±SD)	2.7±1	2.6±1	2.5±1

^a,b^ Unpaired t-test, 1 *df*, *P*<0.0001.

For the ICSI/PGTA cohort, ejaculates were processed in the standard fashion, yielding a sperm concentration of 47.8±14x10^6^/mL with 87.9±12% motility. In the 105 couples included in the ICSI/PGTA+GS cohort, 59 desired a female child, while 46 desired a male child. Sperm sex enrichment for females resulted in a somewhat decreased concentration of 16.7±15x10^6^/mL, with an improved motility of 88.5±11% (*P*<0.0001). For the 46 couples desiring male children, sex selection sperm processing yielded a concentration of 27.0±25 x10^6^/mL (*P*<0.0001) with 83.8±14% motility. Sperm morphology remained consistent in the experimental cohorts in relation to the control (Table 1).

We then proceeded to evaluate the effectiveness of our sex selection method by assessing the sex proportion of spermatozoa from processed specimens (Table 2).

**Table 2:** Proportion of sperm cells enrichment and resulting embryos for female and male sex.

	ICSI/PGTA	ICSI/PGTA+GS	*P* value*
**Female Sex**			
Proportion of 23,X Spermatozoa (%±SD)	49.7±2	81.6±1	<0.0001
Proportion of Female Conceptuses (%)	5084/9305 (54.6)	231/292 (79.1)	<0.00001
**Male Sex**			
Proportion of 23,Y Spermatozoa (%±SD)	50.0±2	80.8±2	<0.0001
Proportion of Male Conceptuses (%)	4221/9305 (45.4)	223/280 (79.6)	<0.00001

*Unpaired t-test, 1 *df*.

**Fig 4:**
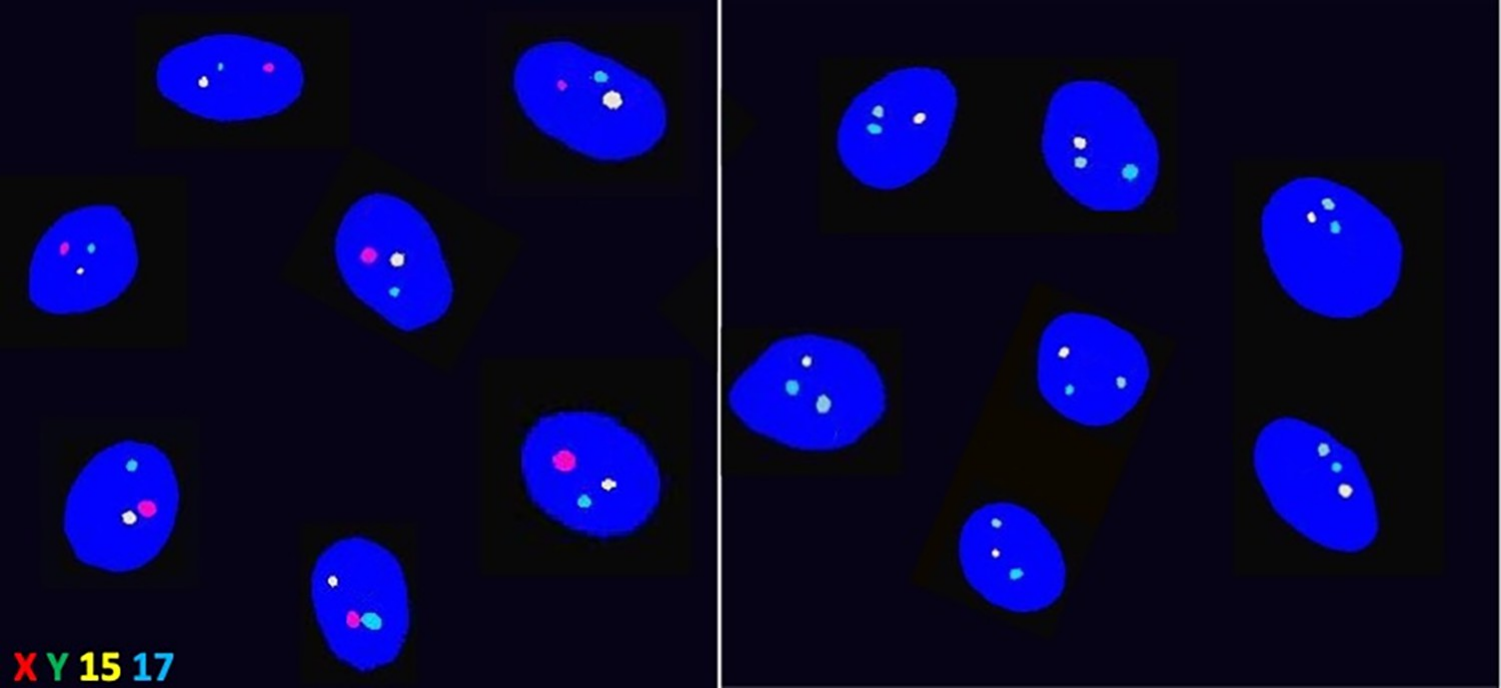
FISH assessment. For couples selecting for female sex, our processing method was able to yield an increased proportion of X-bearing spermatozoa (left). Similarly, our method yielded a greater proportion of Y-bearing spermatozoa for couples selecting for male sex (right).

FISH determined an even proportion of X- and Y-bearing spermatozoa in the ICSI/PGTA group. For couples selecting for female sex, our processing method was able to yield an increased proportion of X-bearing spermatozoa (*P*<0.0001). Similarly, our method yielded a greater proportion of Y-bearing spermatozoa for couples selecting a male sex (*P*<0.0001) (Fig 4).

Next, we assessed the safety of our method by comparing clinical outcomes between the ICSI/PGTA and ICSI/PGTA+GS cohorts (Table 3).

**Table 3:** ICSI outcomes by sex preference.

		ICSI/PGTA+GS	
	ICSI/PGTA	Female	Male
***Couples/ICSI Cycles***	1212/2303	59/73	46/50
Oocytes Retrieved	22354	971	782
MII (%)	17737 (79.3)	754 (77.7)	613 (78.4)
2PN (%)	14375 (81.0)	583 (77.3)	462 (75.4)
Cycles with ET	915	29	21
Embryos Assessed	9305	231/292 (79.1)	223/280 (79.6)
Embryos Transferred	915	29	21
Implantation (%)	699/915 (76.4)	23/29 (79.3)	19/21 (90.5)
Clinical Pregnancy (+FHB) (%)	597/915 (65.2)	18/29 (62.1)	14/21 (66.7)
Deliveries (%)	551/915 (60.2)	16/29 (58.6)	13/21 (61.9)
Pregnancy Loss	46	2	1
***Offspring Delivered***	553	16	13
***Offspring Sex Delivered (F*:*M)***	268:285	16:0	0:13

+FHB: Presence of at least one fetal heartbeat.

ICSI/PGTA couples underwent 2,303 cycles, in which an average of 7.7 oocytes were injected, yielding an 81.0% (14,375/17,737) fertilization rate. In addition, 45.3% (n = 599) of their embryos were female, and 54.7% (n = 724) were male. Following embryo replacement, these couples achieved a 76.4% (699/915) implantation rate and a 65.2% (597/915) clinical pregnancy rate, resulting in 551 deliveries (48.5% female, 51.5% male) and 46 incidences of pregnancy loss.

The 59 couples who wanted female offspring were treated in 73 ICSI cycles and obtained a 77.3% (583/754) fertilization rate. Overall, 79.1% (231/292) of the tested embryos were female. These couples achieved a 79.3% (23/29) implantation rate and a 62.1% (18/29) clinical pregnancy rate, which yielded 16 singleton deliveries of the desired female sex without major or minor congenital malformations.

The 46 couples desiring male offspring were treated in 50 ICSI cycles, which yielded a 75.4% (462/613) fertilization rate and a 79.6% (223/280) proportion of male embryos. They achieved a 90.5% (19/21) implantation rate and a 66.7% (14/21) clinical pregnancy rate, yielding 13 singleton deliveries of healthy baby boys. All children have been faring well, with no developmental delays at 3 years of age. Overall, there were no significant differences among the cohorts in terms of fertilization, implantation, or clinical pregnancy rates.

To further address safety concerns with the use of this sex selection method, we studied the ploidy of the tested embryos of the control (ICSI/PGTA) and study (ICSI/PGTA+GS) groups (Table 4).

**Table 4:** Ploidy of tested embryos for the control and study groups according to sex.

		ICSI/PGTA+GS	
No of Embryos (%)	ICSI/PGTA	Female	Male	
Tested	9305	292	280	
Gender Distribution (F:M)	5084(54.6):4221(45.4)	231(79.1):61(20.9)	57(20.4):223(79.6)	
Euploid Gender Distribution (F:M)	3718:3054	182:47	38:148	

In the ICSI/PGTA cohort, functioning as a reference, there was a comparable sex distribution of embryos observed, where 73.1% of female embryos and 72.4% of male embryos were euploid. Couples in the study group who selected for female obtained a higher proportion of female conceptuses (79.1%), of which 78.8% were euploid. Couples who selected for male sex obtained a higher proportion of male embryos at 79.6%. Of these, 66.4% were euploid. Our comparison did not indicate any significant differences in embryo ploidy according to whether sperm sex selection was performed.

## Discussion

Since the advent of ART, many attempts have been made to accurately select offspring sex. However, most of the techniques that have been used are cumbersome, ineffective, and/or raise safety concerns. The ethics of embryo sex selection is an ongoing debate, but we believe that sex selection of spermatozoa is more ethically acceptable. However, evidence of clinical efficacy and safety is crucial. Therefore, in this study, we included a control group consisting of couples undergoing ICSI/PGTA for reasons other than sex selection.

The current literature on sperm sex selection is limited by the lack of studies that simultaneously assess a method’s technical efficacy, clinical reliability, and safety. Moreover, most sex selection studies are performed in livestock, with many yielding modest or inconsistent results. For instance, a study carried out on bovine spermatozoa processed by the Percoll method excluded a confirmatory FISH sperm assessment and did not appear to evidence sex-enriched embryos [37]. In one of the few studies done on humans, FISH assessment of spermatozoa processed by discontinuous albumin gradients found that specimens were not enriched for sex as previously described [38]. A retrospective cohort study was later carried out exclusively on couples undergoing IVF with PGT-A; however, it did not find any improvement on percentages of male or female embryos [18].

To address the questions left unanswered by previous studies, we aimed to carry out a thorough assessment of our sex selection method. Upon FISH comparison of the processed sperm specimens from the control and study cohorts, we found that our method was consistently able to enrich spermatozoa according to gonosome. We then carried out an assessment to confirm the clinical efficacy of these sex-enriched sperm specimens, and observed that the proportion of tested embryos maintained the sex enrichment.

Following these encouraging findings, we aimed to evaluate the clinical performance of the spermatozoa by comparing ICSI outcomes of the ICSI/PGTA+GS cohort to those from the control group. A recent meta-analysis performed on data obtained from bovine sperm sex selection studies demonstrated that the overall pregnancy and calving rates decreased in cases where sexed sperm specimens were used [39]. Interestingly, a closer analysis found that while the overall stillbirth rate was unaffected by using sexed sperm specimens, stratifying by sex showed a significantly higher incidence of stillborn male calves.

To determine whether our sex selection method affects reproductive outcome, we compared the ICSI results between cohorts, stratifying by the couples’ desired offspring sex. Fertilization, implantation, clinical pregnancy, and delivery rates were not significantly impaired.

The last, and most important, aspect of our assessment is on the safety of our proposed method. Previous studies, particularly those on flow cytometry, have expressed concern about the mutagenic effects of the UV light and DNA binding agent [40]. Although these methods have since been discontinued, the potential risks associated with other sex selection methods remain unclear due to the lack of relevant research. Therefore, we compared embryo aneuploidy rates between the two cohorts. We did not observe a significant increase in embryo aneuploidy, indicating that our method can be safely used to increase the proportion of embryos for a specific sex. For consenting couples, we also assessed the sperm aneuploidy and found that it was unaffected.

In this study, we propose a novel and safe sex selection method that is able to consistently skew the proportion of spermatozoa toward the desired sex for couples undergoing ICSI. Along with the medical rationale of avoiding sex-linked diseases, the scientific rationale for developing our sperm-based sex selection technique is to provide a more ethical procedure to successfully skew the proportion of conceptuses towards a couple’s desired sex without impairing embryo developmental competence or offspring health. Nevertheless, this study is not without limitations. While we were successfully able to maintain the sex enrichment achieved in spermatozoa, embryos were still arbitrarily selected. Thus, a prospective randomized study should be performed as a follow-up assessment to confirm findings. In addition, despite the efficacy demonstrated, there were rare occurrences where all tested conceptuses were of the opposite sex than what we selected for. This may be attributed to subtle morphological and functional differences between the X- and Y-bearing spermatozoa post-selection. Although we did not observe any differences in overall sperm morphology between the cohorts, we cannot exclude that these morphological properties may have influenced the ICSI operator to obviate the sex enrichment by choosing the best-looking spermatozoon at the time of injection.

Although our sperm sex selection method does not necessarily guarantee offspring of a specific sex, couples participating in the study were nonetheless able to obtain a greater proportion of spermatozoa and conceptuses of their desired gonosomal component. Comparisons of ICSI clinical outcomes also showed that fertilization rates and embryo developmental competence were not impaired by using sex-enriched sperm specimens. Moreover, offspring health was not negatively affected. These encouraging findings indicate that not only is our method effective, but also safe, rendering it a feasible and ethically palatable procedure.

## Conclusions

Although ethically debatable, expressing a sex preference for offspring is popular among couples, and not limited to those undergoing infertility treatment. Sperm sex enrichment, within a protocol of PGT-A, enables the selection of embryos for the desired sex. Our sex selection method does not increase the proportion of additional aneuploid embryos. Therefore, it can be regarded as extremely safe as well as efficient, inexpensive, and ethically palatable.

## Supporting information

S1 Checklist TREND statement checklist. (PDF)

S1 File Cycle outcomes for ICSI/PGTA and ICSI/PGTA+GS cohorts. (XLSX)

S2 File (DOCX)
