# Supplementary material for: A non-randomized clinical trial to determine the safety and efficacy of a novel sperm sex selection technique
Source: PLoS One. 2023 Mar 22;18(3):e0282216. doi: 10.1371/journal.pone.0282216 (PMC10032484; doi:10.1371/journal.pone.0282216)
Supplement: S2 File — (DOCX) [file pone.0282216.s003.docx]

**TITLE:** Sex Selection of human spermatozoa

**IRB Protocol #: 1306014043**

**Version Date: 7-12-2022**

**Principal Investigator:** Gianpiero Palermo, MD, PhD

Director, Andrology and Assisted Fertilization

Professor of Reproductive Medicine

Weill Cornell Medicine

1305 York Ave, Suite 702

New York, NY 10021

gdpalerm[@med.cornell.edu](mailto:nizanin@med.cornell.edu)

Tel: 646-962-3689

Fax: 646-962-0344

**Participating Sites:**

Weill Cornell Medicine

Ronald O. Perelman and Claudia Cohen Center for Reproductive Medicine

1305 York Avenue, 6th Floor

New York, NY 10021

Table of Contents

[List of Abbreviations 3](#_Toc3227200)

[1. Study Objectives 4](#_Toc3227201)

[1.1 Objectives 4](#_Toc3227202)

[1.2 Hypotheses / Research Questions 4](#_Toc3227203)

[2. Study Design and Rationale 4](#_Toc3227204)

[2.1 Study Design 4](#_Toc3227205)

[2.2 Rationale and Justification 4](#_Toc3227206)

[2.3 Study Population 6](#_Toc3227207)

[2.4. Statistical Considerations 6](#_Toc3227208)

[3. Data Collection 6](#_Toc3227209)

[3.1 Protected Health Information (PHI) 6](#_Toc3227210)

[3.2 Informed Consent 7](#_Toc3227211)

[3.3 Data Collection/Protection 7](#_Toc3227212)

[3.4 REDCap 7](#_Toc3227213)

[4. Tissue/Blood Usage 7](#_Toc3227214)

[5. Collaboration 7](#_Toc3227215)

[5.1 Internal Collaboration 7](#_Toc3227216)

[5.2 External Collaboration 7](#_Toc3227217)

[6. Additional Information 7](#_Toc3227218)

# List of Abbreviations

*All abbreviations used throughout the protocol must be defined. Add additional abbreviations specific to protocol.*

| **AE** | Adverse Event |
| --- | --- |
| **CFR** | Code of Federal Regulations |
| **CRF** | Case Report Form |
| **CTSC** | Clinical Translational Science Center |
| **DSMB** | Data Safety Monitoring Board |
| **DSMP** | Data Safety Monitoring Plan |
| **FDA** | Food and Drug Administration |
| **GCP** | Good Clinical Practice |
| **HIPAA** | Health Insurance Portability and Accountability Act of 1996 |
| **HRBFA** | Human Research Billing Analysis Form |
| **HUD** | Humanitarian Use Device |
| **ICF** | Informed Consent Form |
| **IDE** | Investigational Device Exemption |
| **IND** | Investigational New Drug |
| **IRB** | Institutional Review Board |
| **PHI** | Protected Health Information |
| **PI** | Principal Investigator |
| **REDCap** | Research Electronic Data Capture |
| **SAE** | Serious Adverse Event |
| **SUSAR** | Suspected Unexpected Serious Adverse Reaction |
| **UIRTSO** | Unanticipated Problem Involving Risks to Subjects or Others |
| **WCM** | Weill Cornell Medicine |

# 1. Study Objectives

## 1.1 Objectives

The primary objective of the study is to demonstrate the efficiency and reliability of selecting gender specific spermatozoa

##

## 1.2 Hypotheses / Research Questions

# 2. Study Design and Rationale

## 2.1 Study Design

Our study aim is to test a simple, reliable, and inexpensive method to attempt selection

of gender specific spermatozoa. Couples will undergo evaluation and treatment according to the standard clinic procedures. Sperm samples provided for infertility treatment will be further analyzed routine semen analysis for volume, concentration, mobility and morphology. Sperm samples will be provided by consenting males undergoing infertility treatment. We will perform a multilayer density gradient utilizing an FDA approved solution, Enhance-S Plus Cell Isolation Media (Vitrolife, San Diego, CA).

This density gradient is also used for our standard semen preparation for intrauterine

insemination. X-bearing spermatozoa and Y-bearing spermatozoa will be identified

from the density gradient fractions. Sperm suspensions will be smeared on slides for

FISH analysis using centromeric probes for chromosomes 18, X, and Y. The ratio of X- to

Y- chromosome bearing spermatozoa will be assessed as a percentage on at least 200

cells per slide. Aneuploid cells and those without signals will be omitted. Unselected

fractions of each sample will serve as controls. The rate of X-bearing spermatozoa

after 4-layer density gradient will be calculated. After thorough counseling, the

selected preconception gender specimen will then be utilized for assisted reproductive

fertilization- in vitro fertilization with or without intracytoplasmic sperm injection

(ICSI) or intra uterine insemination which are routine standard of care procedures.

**Inclusion Criteria:**

Couples undergoing infertility treatment with IVF or insemination seeking gender specific offspring for medical and non medical reasons

**Exclusion Criteria:**

Severe male factor

## 2.2 Rationale and Justification

Gender preselection has enormous implications for both animal and human reproduction. The human X chromosome is considerably larger than the Y, and thus the total DNA content of the haploid genome of an X-bearing spermatozoon is about 2.8% greater than that of a Y-bearing spermatozoon. The X chromosome's larger amount of DNA also enables it to carry numerous X-linked genes, many of which are of fundamental biological importance. Defective function of X-linked genes causes a wide variety of X-linked genetic diseases, of which haemophilia, Duchenne muscular dystrophy, and fragile X syndrome are prominent examples. In general, X-linked disorders are expressed only in males, and female carriers are protected from disease expression by the paired normal allele on their second X chromosome. Such carrier females transmit the genetic disorder to half, on average, of their male offspring. Preconception gender selection finds one important application in reducing the risk of having male children afflicted with a serious or fatal X-linked genetic abnormality.

The first and most widely used method for pre-conceptual gender choice of human babies was that of Ericsson (Ericsson et al., 1973; Beernink et al., 1993). The method was based on the passage of spermatozoa through columns of human serum albumin (HSA), and was founded upon the idea that Y-bearing spermatozoa swim faster than X-bearing spermatozoa. Passage through Percoll has also been used (Batzofin et al., 1995) as has a swim-up procedure (Check and Katsoff, 1993). These last two procedures, however, have not been confirmed by others. In our laboratory, we have attempted to perform hyperosmotic swelling followed by gel trapping (Kan et al., 2000) or by 2D gel electrophoresis (Bartsich et al., 2001). Flow cytometric separation of X- and Y-bearing spermatozoa was achieved in animals (Johnson et al., 1989) and led to weighting of the sexes in the animals’ offspring. The same method was used to separate human X- and Y-bearing spermatozoa (Johnson et al., 1993), and has been used more recently to determine the female sex in preventing X-linked disease (Levinson et al., 1995). However, flow cytometry is subject to the theoretical risks of exposure to the vital dye fluorochrome Hoechst 33342 and to a UV laser beam. Microsort (Schulman and Karabinus, 2005), the most successful method to date, claims to achieve a true enrichment of X-bearing cells at approximately 90%, but requires a major equipment investment and raises additional concerns about exposing sperm DNA to fluorescent staining and UV excitation.

Bartsich S, Hariprashad JJ, Rosenwaks Z, Palermo GD. (2001) Gender selection of human spermatozoa: a simple and effective approach. (Abstr. P-091). In the abstract book of the 17th Annual Meeting of the European Society of Human Reproduction and Embryology. July 1-4, 2001, Lausanne, Switzerland.

Batzofin, J., Tran, C., Tan, T. et al. (1995) XY sperm separation for sex selection. Experience with a Percoll gradient technique in a clinical setting. Fertil. Steril., A28.

Beernink, F.J., Dmowsk, W.P. and Ericsson, R.J. (1993). Sex preselection through albumin separation of sperm. Fertil. Steril., 59, 382–386.

Check, J.H. and Katsoff, D. (1993) A prospective study to evaluate the efficacy of modified swim-up preparation for male sex selection. Hum. Reprod., 8, 211–214.

Cheung S, Neri QV, Husserl PJ, Rosenwaks Z, Palermo GD. (2013) An inexpensive method for gender pre-selection (Abstr. P-588) In the abstract book of the 69th Annual Meeting of the American Society for Reproductive Medicine to be held conjoint with the International Federation of Fertility Societies, Fertility and Sterility. October 12-17, 2013, Boston, Massachusetts, USA.

Ericsson, R.J., Langevin, C.N. and Nishino, M. (1973) Isolation of fractions rich in human Y bearing sperm. Nature, 246, 421–424

Johnson, L.A., Flook, J.P. and Hawk, H.W. (1989) Sex preselection in rabbits: live births from X and Y sperm separated by DNA and cell sorting. Biol. Reprod., 41, 199–203.

Johnson, L.A., Welch, G.R., Keyvanfar, K. et al. (1993) Gender preselection in Humans? Flow cytometric separation of X and Y spermatozoa for the prevention of X-linked disease. Hum. Reprod., 8, 1733–1739.

Kan MT, Hariprashad JJ, Akerman A, Rosenwaks Z, Palermo GD. (2000) Y-bearing enrichment of human spermatozoa by hyperosmotic centrifugation with gel trapping. (Abstr. P-181). In the abstract book of the 56th Annual Meeting of the American Society for Reproductive Medicine, Fertility and Sterility. October 21-26, 2000, San Diego, California, USA. Abstract was selected for Preliminary Poster Award

Levinson, G., Keyvanar, K., Wu, J.C. et al. (1995) DNA-based X-enriched sperm separation as an adjunct to preimplantation genetic testing for the prevention of X-linked disease. Mol. Hum. Reprod., 10 979–982.

Schulman JD, Karabinus DS. (2005) Scientific aspects of preconception gender selection. Reprod Biomed Online., 10, 111-115.

## 2.3 Study Population

Subjects are health males and females who are undergoing infertility treatment. Subjects are not seriously of terminally ill.

## 2.4. Statistical Considerations

The total number of subjects recruited at WCMC will be 2000 with the expected

screen failure rate of about 20% due to drop out and inadequate samples. We plan to recruit 1000 couples (2000 subjects) for this research project.

# 3. Data Collection

## 3.1 Protected Health Information (PHI)

For the study, we will review medical history, embryology and medical information to include demographic Information, medication information, blood test results, ultrasound scan results, physical Examination Information and treatment outcomes. Data is recorded both electronically and in paper format and will be saved on a secure server on a password-protected computer or in a locked office. At the end of the study electronic identifiers will be deleted from the database and paper identifiers will be shredded.

## 3.2 Informed Consent

Informed consent will be obtained form both male and female partners in the clinical office of the Center for Reproductive Medicine by one of the fertility physicians or the research staff during their consultation. Subjects will be asked if they are interested in having their gametes (eggs and sperm) treated to help increase the chances of fertilization, particularly due to their previous history of failed fertilization with ICSI.

Potential subjects will be provided with study information and consent documents and will be given enough time to review the information and ask questions of the researchers.

## 3.3 Data Collection/Protection

All data will be saved electronically on a secure server or password-protected computer that only the study team will have access to.

## 3.4 REDCap

N/A

# 4. Tissue/Blood Usage

N/A

# 5. Collaboration

## 5.1 Internal Collaboration

Ronald O. Perelman and Claudia Cohen Center for Reproductive Medicine

## 5.2 External Collaboration

*N/A*

# 6. Additional Information

*N/A*
